# Temperature sensitive influenza A virus genome replication results from low thermal stability of polymerase-cRNA complexes

**DOI:** 10.1186/1743-422X-3-58

**Published:** 2006-08-25

**Authors:** Rosa M Dalton, Anne E Mullin, Maria Joao Amorim, Elizabeth Medcalf, Laurence S Tiley, Paul Digard

**Affiliations:** 1 Division of Virology, Department of Pathology, University of Cambridge, Tennis Court Road, Cambridge CB2 1QP, UK; 2 Centre for Veterinary Science, University of Cambridge, Madingley Road, Cambridge CB3 OES, UK

## Abstract

**Background:**

The RNA-dependent RNA polymerase of Influenza A virus is a determinant of viral pathogenicity and host range that is responsible for transcribing and replicating the negative sense segmented viral genome (vRNA). Transcription produces capped and polyadenylated mRNAs whereas genome replication involves the synthesis of an alternative plus-sense transcript (cRNA) with unmodified termini that is copied back to vRNA. Viral mRNA transcription predominates at early stages of viral infection, while later, negative sense genome replication is favoured. However, the "switch" that regulates the transition from transcription to replication is poorly understood.

**Results:**

We show that temperature strongly affects the balance between plus and minus-sense RNA synthesis with high temperature causing a large decrease in vRNA accumulation, a moderate decrease in cRNA levels but (depending on genome segment) either increased or unchanged levels of mRNA. We found no evidence implicating cellular heat shock protein activity in this effect despite the known association of hsp70 and hsp90 with viral polymerase components. Temperature-shift experiments indicated that polymerase synthesised at 41°C maintained transcriptional activity even though genome replication failed. Reduced polymerase association with viral RNA was seen *in vivo *and in confirmation of this, in *vitro *binding assays showed that temperature increased the rate of dissociation of polymerase from both positive and negative sense promoters. However, the interaction of polymerase with the cRNA promoter was particularly heat labile, showing rapid dissociation even at 37°C. This suggested that vRNA synthesis fails at elevated temperatures because the polymerase does not bind the promoter. In support of this hypothesis, a mutant cRNA promoter with vRNA-like sequence elements supported vRNA synthesis at higher temperatures than the wild-type promoter.

**Conclusion:**

The differential stability of negative and positive sense polymerase-promoter complexes explains why high temperature favours transcription over replication and has implications for the control of viral RNA synthesis at physiological temperatures. Furthermore, given the different body temperatures of birds and man, these finding suggest molecular hypotheses for how polymerase function may affect host range.

## Background

The genome of influenza A virus consists of eight single-stranded, negative-sense genomic RNA (vRNA) segments, associated with nucleoprotein (NP) and the viral polymerase complex (PB1, PB2 and PA) in the form of ribonucleoprotein complexes (RNP) [1]. The viral RNA-dependent RNA polymerase is responsible for both transcription and replication of the viral genome, which take place in the cell nucleus [2,3]. The first step in viral gene expression is the transcription of the incoming vRNPs into mRNA, through a primer-dependent process in which 5'-capped RNA fragments of 10–15 nt are cleaved from host-cell pre-mRNAs and used as primers [4]. The resulting transcripts are polyadenylated at their 3' end when the polymerase, reiteratively incorporates A residues as a result of stalling at the polyuridine stretch [5-7] immediately adjacent to the 5' terminus of vRNA, to which the polymerase remains bound. These mRNA transcripts, being incomplete copies of the vRNA template, cannot serve as substrates for replication of new vRNA molecules. Viral genome replication is primer independent and generates full-length positive-sense cRNA transcripts, that are not polyadenylated [8-10]. This replicative intermediate subsequently serves as the template for synthesis of progeny vRNA.

The temporal pattern of RNA production is well established [11-15]. The replicative intermediate, cRNA, is first detected during the early stages of viral infection, reaches a maximum rate of synthesis prior to that of m- or vRNA and then declines. Maximal rates of mRNA synthesis also occur relatively early, before substantial amounts of vRNA are made. Amplification of vRNA continues even after m- and cRNA levels decline. Generally therefore, the early stages of viral infection can be defined by a prevalence of plus sense transcription, comprised mostly of mRNA with a minority of cRNA, while at later times negative sense replication in the form of vRNA synthesis is favoured. Various models have been proposed to explain the "switch" that regulates the transition from transcription to replication. De novo protein synthesis, and therefore a first round of viral transcription, is necessary for genome replication to occur [11,13]. NP is undoubtedly required for both positive and negative sense genome replication [10,16], but its precise role is not known [17-20]. Newly synthesized polymerase protects the termini of cRNA molecules from nuclease attack [21], which at least partly explains its requirement for the accumulation of cRNA. Some studies support the hypothesis that alternative forms of the polymerase catalyse transcription and replication, with a dimeric PB1-PA complex perhaps being the minimum requirement for genome replication [22,23]. However, this is controversial with several other groups finding an essential role for PB2 in transcription and replication [24-26]. Host cell polypeptides may also be involved in the shift to genome replication [27-30], but this is uncertain.

Understanding the determinants of species tropism for influenza virus has never been more important than it is now, with the concern about the potential of avian H5N1 virus to adapt to human hosts. While tropism is a multifaceted and complex process, it has long been hypothesized that something as simple as the temperature at the site of replication could influence the host and tissue tropism of the virus [31-33]. Human-tropic influenza viruses are considered to replicate in the upper respiratory tract at 33–37°C, while avian influenza viruses replicate in the gut around 41°C [32]. The polymerase is an important determinant of influenza virus host range and pathogenicity [34-39] that is likely to be influenced by temperature. Firstly it is a multifunctional enzyme, and secondly, the consequence of its interaction with the 5' and 3' termini of viral RNA is modulated by whether these regions are in a base-paired or single stranded conformation [40]. We therefore examined the temperature dependency of polymerase function for mammalian adapted virus strains in cell culture and in vitro assay systems. We found that vRNA synthesis was markedly reduced at elevated temperatures, whereas mRNA synthesis was stimulated. We found no evidence to implicate heat shock proteins in this temperature effect, despite their known interaction with influenza A RNPs [30,41]. The reduction of vRNA synthesis correlated with a markedly increased dissociation rate of the viral polymerase from cRNA at 41°C. Furthermore, we find that at 37°C the interaction of polymerase with cRNA was significantly less stable than with vRNA, a finding with implications for the regulation of viral RNA synthesis and for the adaptation of influenza viruses to host species with different body temperatures.

## Results

### Effect of temperature on viral RNA synthesis

**Figure 1:**
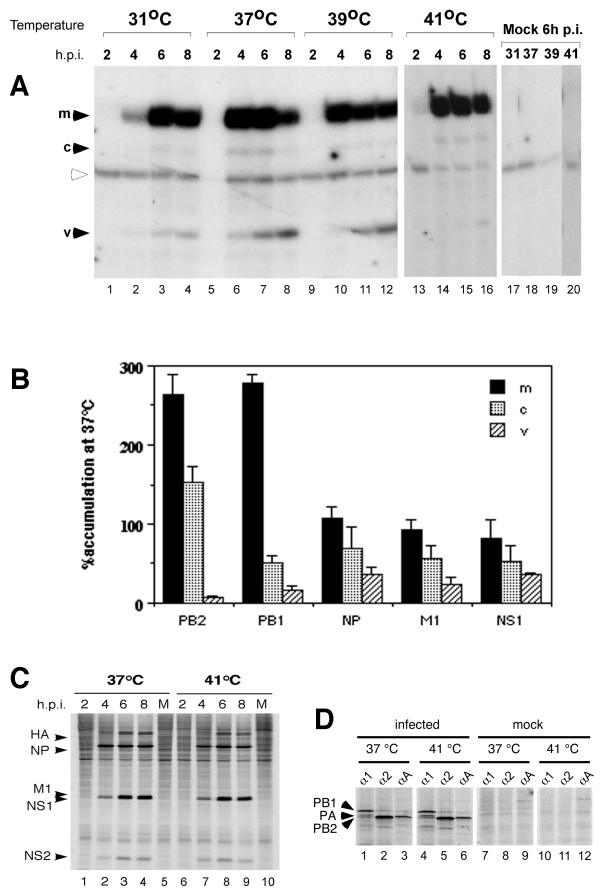
**Effect of temperature on viral RNA and protein synthesis**. 293T cells were infected with PR8 virus or mock-infected (lanes 17–20) and incubated at 31°C, 37°C, 39°C or 41°C. **A**. Total cellular RNA was isolated at the indicated times post-infection and subjected to primer extension analysis for segment 5 transcripts. Products were separated on a 6% polyacrylamide gel and visualised by autoradiography. Black arrows indicate products derived from viral RNAs (as labelled). The white arrow indicates a cellular derived background product. Note the generally lower levels of product in lane 5, due to a loss of that particular sample. **B**. Segment 1, 2, 5, 7 and 8-specific RNA from cells incubated at 37°C and 41°C was quantified at 5 h.p.i. by densitometry of exposed X-ray film. The amounts at 41°C are expressed as the percentage of the corresponding 37°C values. Mean % ± SD from 3 (segment 8), 4 (segment 1) or 5 (segments 2, 5 and 7) independent experiments are plotted. **C, D**. Mock-infected (M) or infected 293T cells, incubated at either 37°C or 41°C, were pulse radiolabelled with 60 nCi/μl ^35^S-Methionine for 2 h periods ending at the indicated times p.i. before analysis by SDS-PAGE and autoradiography or (D) immunoprecipitation using rabbit antiserum against PB1 (α1), PB2(α2) or PA (αA). Black arrows indicate viral proteins (as labelled).

To test the influence of incubation temperature on viral RNA synthesis in the context of virus infection, cells were inoculated with influenza A/PR/8/34 (PR8) virus and incubated at different temperatures. Infected cell lysates were harvested every two hours until eight hours post-infection (h.p.i.), and total cellular RNA was isolated. Reverse transcriptase primer extension analysis using two oligonucleotides to simultaneously detect m-, c- and vRNA [20] was conducted to detect and measure the relative amounts of RNA produced from segment 5 (NP). A primer extension product that presumably resulted from cross-hybridization with a cellular RNA was observed from all samples, infected and mock infected, at all temperatures (Fig. 1A). The three expected virus specific RNA species were synthesised in infected cells at all incubation temperatures. Mock-infected cells did not generate any virus specific products (Fig. 1A, lanes 17–20). At 37°C (lanes 5–8) the time course of viral RNA synthesis reflected that which has been previously described in the literature [11,13,14,20]; early synthesis of m- and cRNA followed by late synthesis of vRNA. At 31°C, there was less replicative RNA synthesis overall than at 37°C and the timing of RNA production appeared to lag by 2 hours (Fig. 1A, compare lanes 2–4 to 6–8). Both m- and vRNA accumulated to equivalent levels with similar timing at 39°C compared to 37°C (Fig. 1A, compare lanes 6–8 to 10–12). However, when infected cells were incubated at 41°C, the levels of segment 5 vRNA were substantially decreased in comparison to those detected at 37°C, while mRNA levels were unchanged (Fig. 1A, compare lanes 6–8 to 14–16). This same trend was seen when radiolabeled primer extension products for five different segments were quantified by densitometry (Fig. 1B). At 41°C on average, segment 5 mRNA accumulation at 5 h.p.i. was unchanged, cRNA accumulation was slightly reduced, but vRNA accumulation dropped by about 3-fold, compared with 37°C incubated cells. Similar results were obtained for segments 7 (M1/M2) and 8 (NS1/NS2). Replication of segments 1 (PB2) and 2 (PB1) was even more sensitive to high temperature, whereas mRNA levels from these two segments were increased more than 2-fold. Thus, at high temperature the polymerase appears to be limited to the early pattern of transcription and unable to switch to the late pattern of negative sense genome amplification. This temperature effect on viral RNA synthesis was also observed for other mammalian influenza strains, such as A/Victoria/3/75 and A/Equine/Miami/63 (data not shown), suggesting it is a general phenomenon for mammalian influenza A virus.

To test if the alteration in transcriptional balance at high temperature resulted from differential expression of the viral polypeptides, protein synthesis was analysed by ^35^S-methionine labelling. Time course analyses of infected cells showed similar patterns of viral protein synthesis at 37°C and 41°C (Fig. 1C, compare lanes 1–4 to lanes 6–9). Similar levels of polypeptides of the predicted size for HA, NP, M1, NS1 and NS2 were detected at both temperatures but were not present in mock-infected cells (Fig. 1C, lanes 5 and 10). Immunoprecipitation assays using antibodies against the three subunits of the viral polymerase also showed similar levels of PB1, PB2 and PA at both temperatures (Fig. 1D, compare lanes 1–3 to 4–6). Furthermore, the levels of co-precipitation of the three P proteins were similar at 41°C and 37°C, suggesting the polymerase complex is still formed at the higher temperature. No specific polypeptides were precipitated from mock-infected cell lysates by any of the three antibodies (lanes 7–12). The overall accumulation of PB1, M1 and NP were also found to be similar at either temperature by western blotting (data not shown). These results are consistent with the undiminished levels of viral mRNA observed at high temperature. Therefore, incubation of infected cells at 41°C does not substantially alter viral protein synthesis and the deficiency of any particular virus protein required for genome replication is an unlikely explanation for defective vRNA synthesis at high temperature.

**Figure 2:**
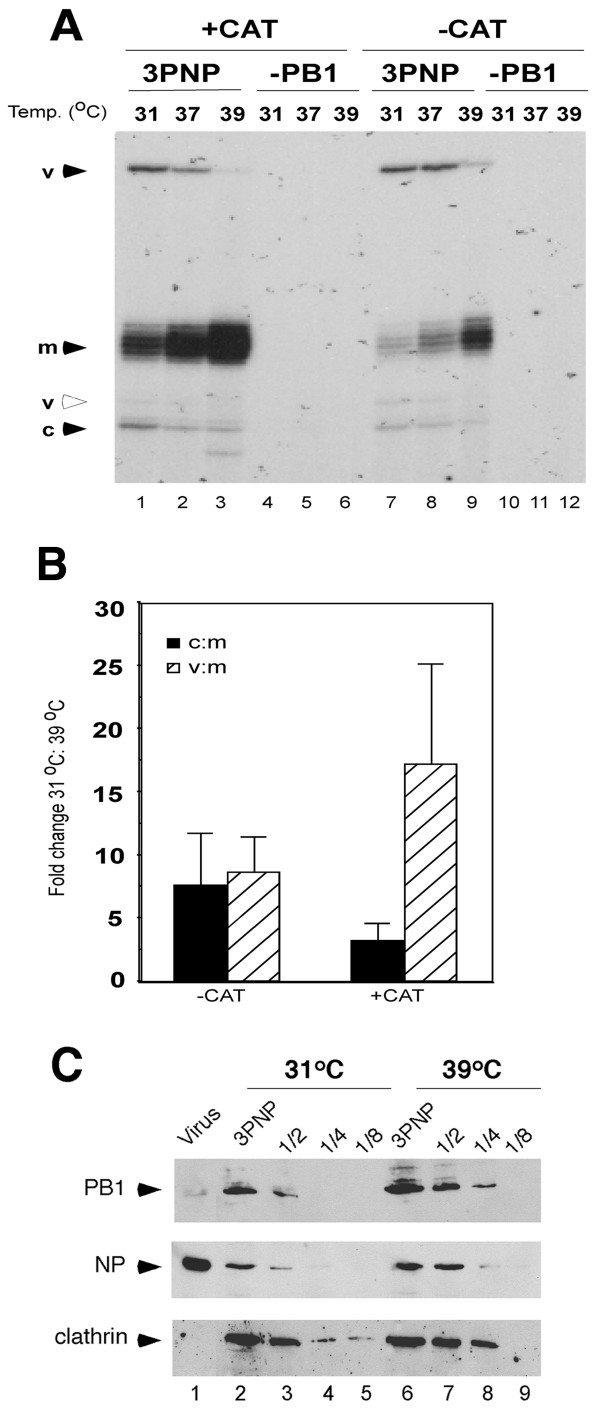
**Effect of temperature on the activity of reconstituted influenza virus RNPs**. 293T cells were transfected with plasmids for the expression of PB1, PB2, PA and NP (3PNP) and either pPol-I(-)NSCAT (-CAT) or pPol-I(+)NSCAT (+CAT) or the same without PB1 (-PB1). Cells were incubated at 31°C, 37°C or 39°C as indicated for three days. **A**. Total cellular RNA was isolated and subjected to primer extension analysis for virus-derived CAT m-, c- and vRNA, as labelled. Products of primer extension analysis were separated by 6% denaturing PAGE and detected by autoradiography. The open arrowhead indicates a truncated product derived from vRNA. **B**. Radiolabeled products for m- c. and vRNA were quantified by densitometry. The ratios of cRNA:mRNA and vRNA:mRNA were calculated and are shown as the fold change (average ± S.D.) in ratios between 31°C and 39°C for cells seeded with vRNA (-CAT) (n = 3) and cRNA (+CAT) (n = 6). **C**. Doubling dilutions (up to 1/8) of total cell lysates were analysed by electrophoresis and western blotting to detect PB1, NP and clathrin. Purified virions were also included to provide a size marker for viral proteins (lane 1).

A plasmid based recombinant system that recreates functional influenza virus RNPs in cells [3,20] was also used to test the influence of temperature on the replication/transcription balance. In this system, synthesis of the viral proteins is driven by the Cytomegalovirus immediately early promoter and is thus uncoupled from the levels of transcription and replication of the viral RNAs. 293T cells were co-transfected with plasmids that separately expressed the three polymerase proteins, NP (all from influenza virus PR8) and a model v- or cRNA segment containing a chloramphenicol acetyltransferase (CAT) gene. Cells were then incubated at 31°C, 37°C or 39°C for three days and total cellular RNA isolated. The viral RNA species synthesized by recombinant RNPs at the different temperatures were analysed by primer-extension assay. No products were observed from cells transfected with all plasmids except PB1 (Fig. 2A, lanes 4–6 and 10–12). When cells expressed the complete set of RNP polypeptides and either the positive or negative-sense model segment, primer extension products of the predicted sizes [20] for viral m-, c-, and vRNA were detected at all incubation temperatures (Fig. 2A lanes 1–3 and 7–9), indicating that the recombinant RNPs were active for both transcription and replication. However, it was clear that as the incubation temperature increased from 31°C to 39°C the amount of replicative RNA products (c- and vRNA) decreased and a greater accumulation of mRNA was observed. This change from replication to transcription with increasing temperature was not altered by the sense of the model virus segment used to seed the reactions, as all three viral RNA species altered in abundance as temperature was varied, with both (+) and (-) CAT-primed reactions. When radiolabeled products were quantified by densitometry in replicate experiments the trend was confirmed (Fig. 2B). In experiments where a negative polarity CAT segment was introduced, the ratios of cRNA to mRNA and vRNA to mRNA changed by around 8 fold across the temperature range. When a cRNA-like CAT RNA was transfected the cRNA:mRNA ratio decreased by over 3-fold and the vRNA:mRNA ratio decreased by 10-fold or greater as incubation temperature was shifted from 31°C to 39°C. Furthermore, the temperature sensitivity was not an artefact of the particular cDNA clones used, as similar effects were noted when RNPs were reconstructed with clones from another human influenza virus, A/Victoria/75, and when authentic influenza segments from A/WSN/33 strain were produced from reverse genetic plasmids (data not shown).

The synthesis of NP and PB1 in transfected cells at the different incubation temperatures was analysed by western blotting. Temperature did not significantly alter the accumulation of either viral protein relative to a constitutively expressed cellular protein not known to be affected by temperature (Fig. 2C compare lanes 2–5 to 6–9). This suggests that similarly to authentic viral infection, alterations to the expression levels of viral proteins do not explain the temperature dependent change in the balance between viral transcription and replication.

Thus, the balance between transcription and replication of the influenza virus genome is affected by temperature in the settings of both infection and transfection, although a wider range of temperature affects the latter system.

### Heat shock proteins as potential factors in the temperature-dependent inhibition of viral genome replication

Heat shock protein (hsp) synthesis and activity plays an important role in the cellular response to stress conditions, such as high temperature [42,43]. Several reports have indicated an interaction between hsps and influenza virus RNP components. Hsp70 has been reported to interact with NP at 41°C and to consequently block vRNP nuclear export [41]. Since NP is also intimately involved in viral RNA synthesis [1] it is possible that this cellular interaction might influence vRNA replication. Similarly, hsp90 has been suggested to bind two or more of the polymerase subunits at 37°C, with possible stimulatory or inhibitory effects on virus RNA synthesis [26,30]. We therefore tested the hypothesis that hsp activity was responsible for the down-regulation of vRNA synthesis at 41°C.

**Figure 3:**
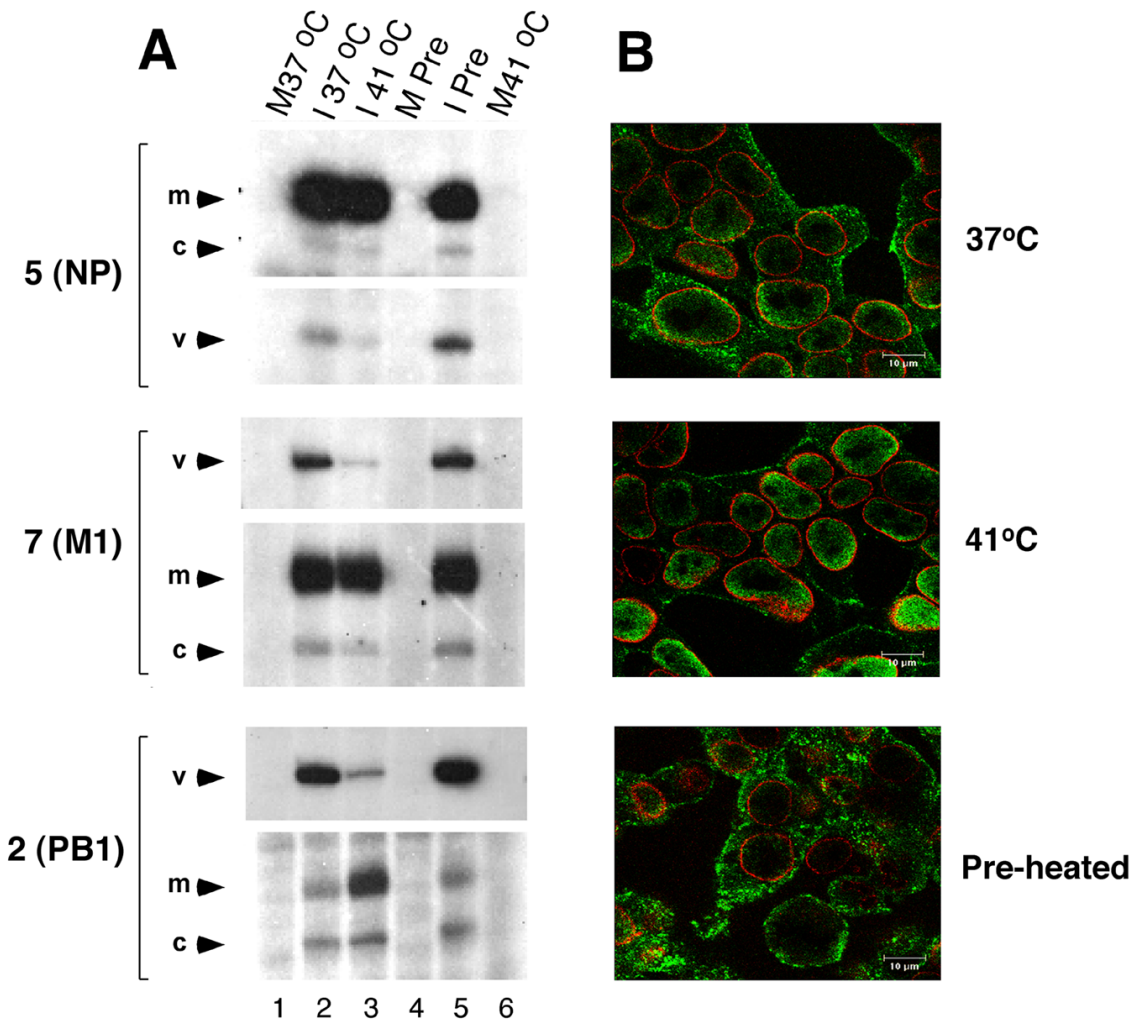
**Effect of pre-heating cells on viral infection**. 293T cells were incubated at 41°C for 4 h prior to infection with PR8 virus (I Pre) or mock-infection (M Pre), and incubated for 5 h at 37°C. Cells infected (I) or mock-infected (M) and incubated at 37°C or 41°C served as controls. **A**. Total RNA was isolated and subjected to radiolabelled primer extension analysis to detect segment 5, 7 and 2-specific RNA. **B**. Cells were fixed at 5 hpi and analysed by confocal microscopy after staining for NP (green) and LAP-2 to delineate the nucleus (red). Bars 10 μm.

First, to test for a correlation between hsp induction and the decrease in viral RNA replication, 293T cells were heated at 41°C for 4 hours to induce hsp synthesis, infected with influenza virus and incubated at 37°C for a further 5 hours. Western blotting confirmed that both hsp70 and hsp90 synthesis were induced by incubation at 41°C and that the same high levels were maintained for at least another 5 hours at 37°C (data not shown, but see later). Non-preheated cells were also infected and incubated at either 37°C or 41°C for 5 hours as controls. Total cellular RNA was isolated and primer extensions performed to analyse viral RNA synthesis from segments 2, 5 and 7. As previously observed, when cells were infected and incubated at 41°C for 5 hours, vRNA accumulation was reduced compared to 37°C (Fig. 3A, lanes 2 and 3). Also as before, NP and M1 mRNA levels were similar in cells incubated at either temperature, while PB1 mRNA was increased at 41°C. However, synthesis of vRNA in the pre-heated cells was comparable to that observed at 37°C (Fig. 3A, compare lanes 5 to 2 and 3). Induction of hsp70 has been associated with the inhibition of RNP nuclear export [41]. However, while immunofluorescence analysis confirmed nuclear retention of NP at 41°C, pre-heated cells presented a cytoplasmic localization pattern for NP, similar to the expected late staining pattern [44] observed in non-preheated cells infected at 37°C (Fig. 3B). Similar results were obtained when the experiments were carried out in MDCK cells (data not shown). Overall, neither genome replication nor RNP nuclear export were inhibited in infections carried out at 37°C, even in the presence of hsps synthesised during a prior heat shock at 41°C. Thus, hsp induction by itself is not sufficient to alter the replication/transcription balance of the virus.

**Figure 4:**
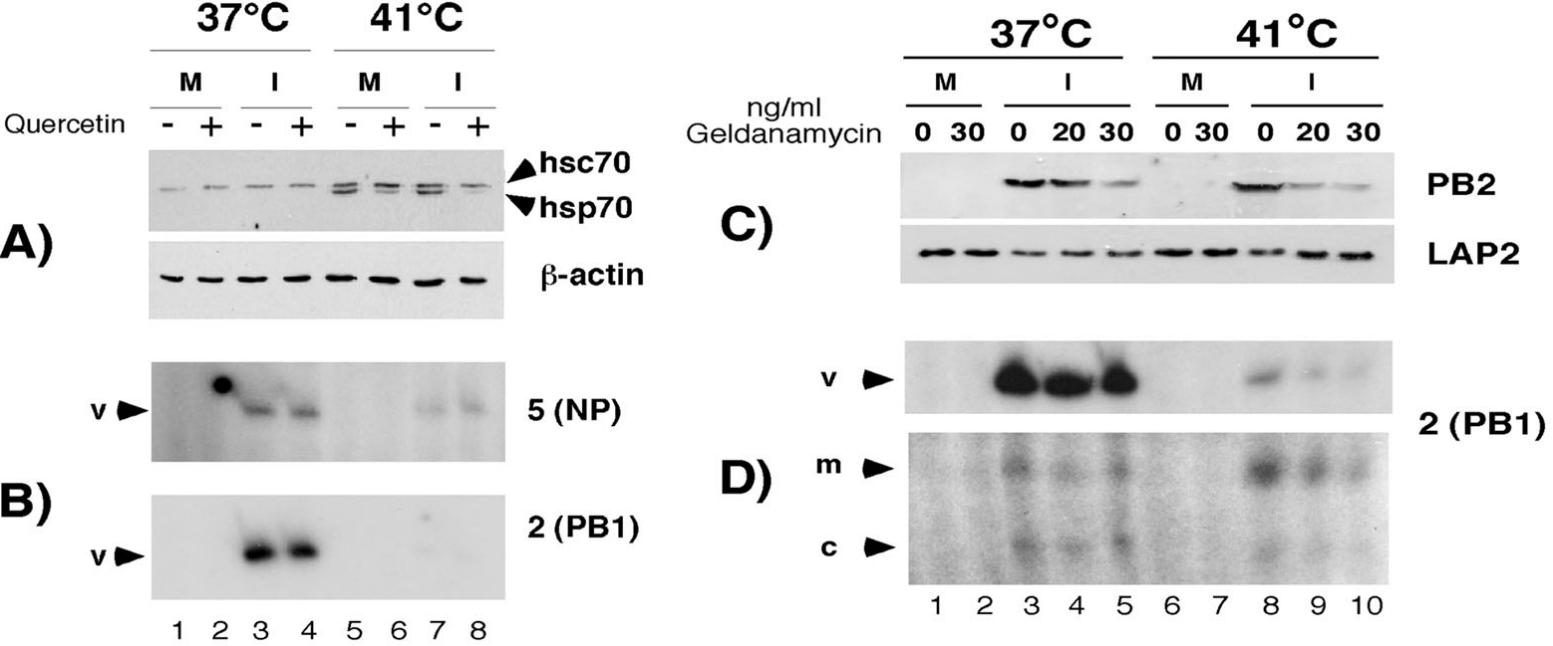
**Effect of chemical inhibition of heat shock responses on viral RNA synthesis**. **A, B**. Inhibition of hsp70 synthesis by quercetin. MDCK cells were infected (I) or mock-infected (M) with PR8 virus, treated (+) or mock-treated (-) with 30 μM quercetin and incubated at 37°C or 41°C for 5 h. **A**. Cell lysates were analysed by SDS-PAGE and Western blotting to detect hsp70/hsc70 and, as a loading control, β-actin. **B**. Total RNA was isolated and primer extension analysis was carried out to detect segment 5 and 2 vRNA. **C, D**. Inhibition of hsp90 chaperone function by geldanamycin. 293T cells were infected (I) or mock-infected (M) with PR8 virus, treated with 0, 20 or 30 ng/ml of geldanamycin (as indicated) and incubated at 37°C or 41°C for 5 h. **C**. Cell lysates were analysed by Western blotting to detect PB2 and, as a loading control, LAP-2 proteins. **D**. Total RNA was analysed by primer extension to detect segment 2-specific RNA.

Although hsp induction does not down-regulate vRNA synthesis at 37°C, a temperature-dependent activity of hsps could be involved. For example, during heat shock hsp90 oligomerizes with the possible activation of functions that are normally silent at physiological temperature [45]. To test this hypothesis two drugs that interfere with hsp responses were used. First, quercetin was used to inhibit hsp70 synthesis [46,47]. MDCK cells were infected and treated or mock-treated with 30 μM quercetin for 5 hours at either 37°C or 41°C. Western blot analysis of hsp70 (using an antibody that recognises the constitutively expressed 72 kDa hsc70 and the 70 kDa inducible hsp70 [43]) showed an induction of this protein at 41°C in both mock-infected and infected cells (Fig. 4A, compare lanes 1 to 5 and 3 to 7). However, hsp70 levels were markedly lower when cells were treated with quercetin compared to untreated cells (compare lane 5 to 6 and 7 to 8). Nevertheless, primer extension analysis for segments 2 and 5 showed that the levels of vRNA accumulation in infected-cells incubated at 41°C remained substantially lower than at 37°C, even with quercetin treatment (Fig. 4B, compare lane 3 to 7 and 4 to 8). The levels of c- and mRNA were also similar in treated and untreated cells (data not shown).

Next, geldanamycin was used to interfere with hsp90 function. Hsp90 participates in two multichaperone complexes with opposing activities depending on the co-chaperone proteins attached to it. In one conformation the hsp90 complex binds to and stabilizes its client proteins, in the other, it promotes client protein ubiquitination and degradation by the proteasome. Geldanamycin binds to hsp90 and forces it to adopt the conformation that favours proteasome-targeting and prevents the stabilization function [48]. 293T cells were inoculated with influenza virus and incubated at either 37°C or 41°C for 5 hours. Cells were either mock-treated or treated with 20 or 30 ng/ml of geldanamycin immediately after the virus adsorption period. Western blot analysis of the cell lysates showed decreasing levels of PB2 as the concentration of geldanamycin was increased at either temperature (Fig. 4C, lanes 3–5 and 8–10). This is consistent with a PB2-hsp90 interaction [30] that in the presence of geldanamycin results in the degradation of the client protein. Primer extension analysis for segment 2 was carried out on total cellular RNA extracted from these samples to test viral RNA synthesis during drug treatment. As previously observed for segment 2, levels of vRNA accumulation were lower, and those of mRNA higher, at 41°C compared to 37°C in non-treated cells (Fig. 4D, compare lane 3 to 8). However, when cells infected at 41°C were treated with either 20 or 30 ng/ml of geldanamycin the levels of vRNA remained low and in fact were further decreased by the drug (compare lanes 4 and 5 to 9 and 10).

Thus, at 41°C viral genome replication cannot be rescued by inhibiting hsp70 synthesis or hsp90 chaperone function, providing no support for the hypothesis that either of these cellular proteins is responsible for down-regulation of vRNA synthesis at high temperature, despite their known interactions with RNP components.

### Analysis of the effect of temperature on RNP formation

**Figure 5:**
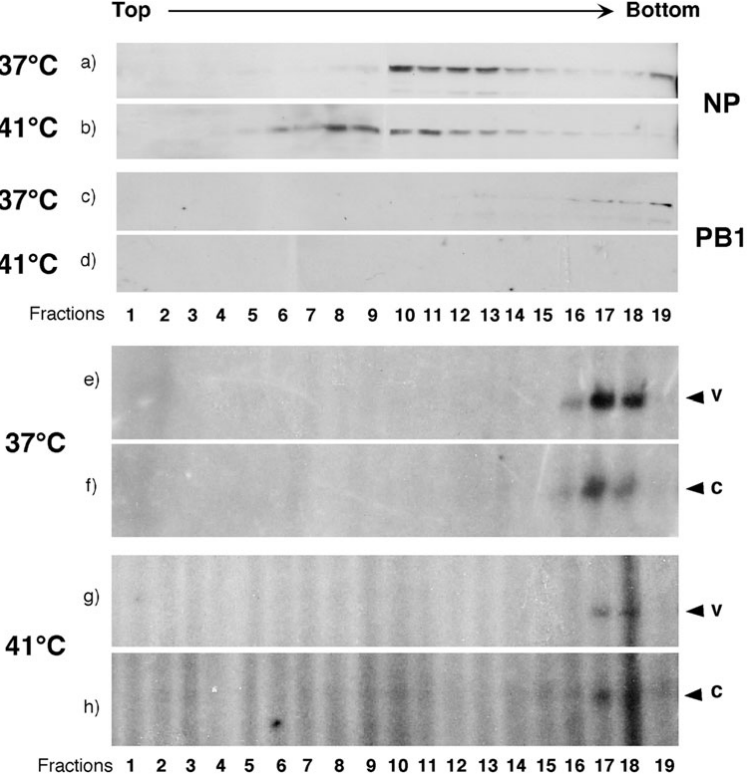
**Analysis of RNP formation at different temperatures**. Infected 293T cells were incubated at 37°C or 41°C for 5 h and lysed as described in Methods. The extracts were layered onto 5–20% sucrose gradients and separated by centrifugation. The resulting fractions were analysed by Western blotting to detect (a, b) NP and (c, d) PB1. (e-h) RNA was also extracted from each fraction and subjected to primer extension analysis to detect segment 7-specific RNA.

A previous study suggested that RNP formation in infected cells was not impaired at 41°C on the basis of the glycerol density gradient centrifugation profile of NP [49]. Although glycerol gradients are often used to purify virion associated RNPs away from membrane and other low density virion components [17] we decided to employ velocity gradients to analyse RNP formation on the grounds that a technique that separates according to molecular weight might provide a more sensitive test. Accordingly, cell lysates prepared from infected cells incubated at 37°C or 41°C were separated by sucrose gradient centrifugation and the individual fractions analysed for protein and RNA content. Western blot analysis of NP showed two peaks from cells incubated at 37°C; a fast sedimenting fraction at the bottom of the gradient and a slower migrating fraction towards the middle (Fig. 5a). When PB1 was examined as a marker for the viral polymerase, this was mostly found as a fast-sedimenting species towards the bottom of the gradient (Fig. 5c). Analysis of segment 7 RNA content showed that these fast-sedimenting pools of PB1 and NP were associated with both v- and cRNA (Fig. 5 e, f). This indicates that these bottom fractions contain RNPs while the slower migrating pool of NP represents unassembled material. Similar results were obtained for segments 2 and 5 except that they sedimented 1 to 3 fractions further down the gradient (data not shown). When material from cells incubated at 41°C was analysed, the amount of fast-sedimenting NP was much reduced and the pool of material around the middle of the gradient was shifted slightly further up the gradient (Fig. 5b). The reduced amounts of fast-sedimenting NP were still associated with detectable v- and cRNA (Fig. 5 g, h, although vRNA in particular was present in much reduced amounts compared to 37°C, consistent with analysis of total RNA content. However, no polymerase was detectable in these high molecular weight fractions (Fig. 5d). Polymerase not assembled onto RNPs was not detected in the slower sedimenting fractions but instead partitioned with insoluble cell nuclear material removed from the lysates by low speed centrifugation before loading the gradient (data not shown). Overall therefore, we conclude there is a defect in vRNA synthesis at 41°C that results in reduced quantities of RNPs that are also deficient in polymerase content.

**Figure 6:**
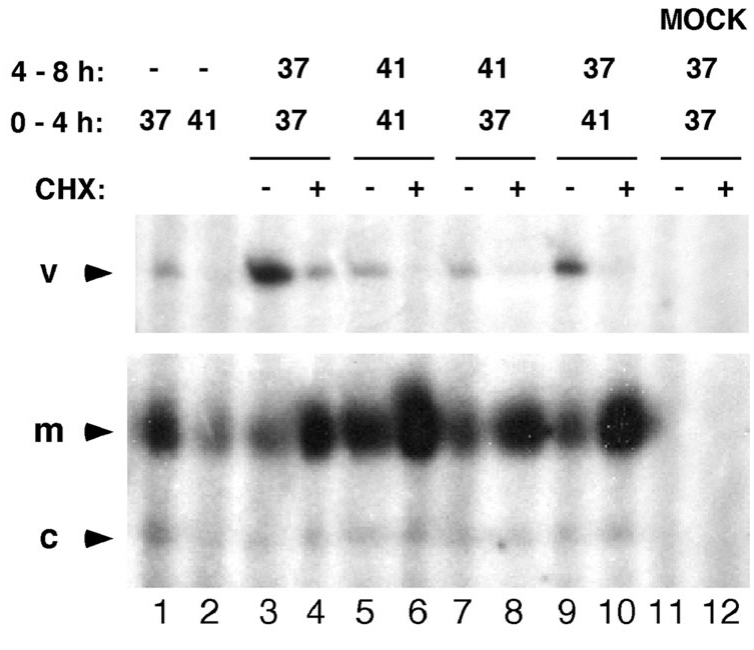
**Effect of temperature on viral polymerase activity**. 293T cells were infected with PR8 virus and incubated at 37°C or 41°C for 4 h. At this point 50 μg/ml of cycloheximide (+) or the same volume of DMSO (-) was added to the cells, temperature was shifted up (lanes 7, 8) or down (lanes 9, 10) and infections left to proceed for another 4 h. Total RNA was isolated and primer extension was carried out to detect segment 7-specific RNAs as labelled. Infected cells incubated at 37°C or 41°C for 4 h (lanes 1, 2) or 8 h (lanes 3, 4 and 5, 6 respectively) served as positive controls. Mock-infected cells incubated at 37°C for 8 h served as a negative control (lanes 11, 12).

Prior work has shown that in vitro, ApG primed transcriptional activity of the polymerase is unstable at 40°C in the absence of vRNA [50]. As our results suggested that the viral polymerase does not remain bound to RNPs at 41°C, we tested the activity of the polymerase under these conditions by temperature shift experiments. Duplicate cell cultures were inoculated with influenza virus and incubated at either 37°C or 41°C for 4 hours. At this point cycloheximide was added to one set of experiments to inhibit protein synthesis and therefore permit the transcriptional activity of polymerase synthesised during the preceding 4 h to be examined in isolation. However, this strategy only works for mRNA synthesis as cycloheximide inhibits genome replication [11,13,51]. The incubation temperature was shifted up or down and infections allowed to proceed for another 4 hours. Control experiments where the temperature was not altered during 8 hours or in which cells were harvested at 4 hours post-infection were also performed. At 8 hours post-infection cell lysates were harvested and segment 7 synthesis examined. No virus-specific products were observed from mock-infected cell lysates (Fig. 6, lanes 11 and 12). At 37°C levels of mRNA decreased and vRNA increased between 4 and 8 h p.i. (Fig. 6, compare lane 1 to 3). At 41°C, mRNA and vRNA levels increased with time (compare lanes 2 and 5) but vRNA accumulation was much decreased compared to 37°C (compare lanes 3 and 5). As previously described [51], when cycloheximide was added after 4 hpi, levels of mRNA were boosted compared to untreated cells and vRNA synthesis was inhibited at either 37°C or 41°C (compare lanes 3 to 4 and 5 to 6). When temperature was shifted up after 4 hpi, accumulation of vRNA stopped (compare lanes 1, 3 and 7). This suggests that polymerase formed at 37°C during the first 4 hours is not capable of synthesising vRNA once placed at 41°C. However, viral RNA synthesis was not globally inhibited as high levels of mRNA were observed in shifted up cells treated with cycloheximide (lane 8). Conversely, when temperature was shifted down after 4 hpi vRNA accumulation recovered (Fig. 6, compare lanes 2, 5 and 9). Similar overall results were observed for segments 2 and 5 (data not shown). Overall, this indicates that at high temperature the viral polymerase is highly transcriptionally active but defective for vRNA synthesis and this temperature dependent switch in activity acts on RNA synthesis itself, not when the polymerase is translated.

### Stability of polymerase-template interactions at elevated temperature

**Figure 7:**
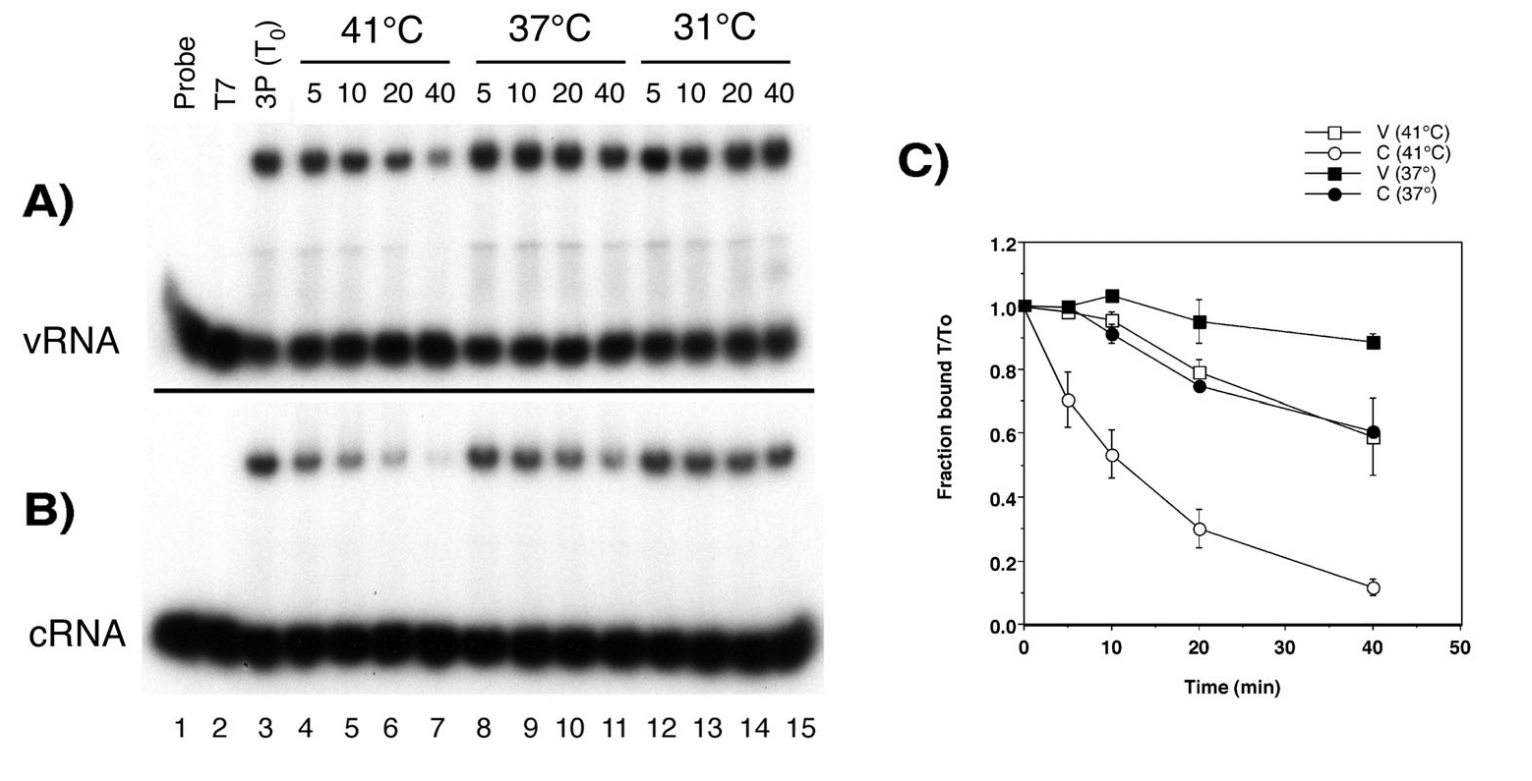
**Effect of temperature on the dissociation of viral polymerase-RNA complexes**. Nuclear extracts containing the bacteriophage T7 RNA polymerase (T7) or the influenza virus polymerase (3P) were bound at room temperature for 10 min to (a) radiolabelled vRNA or (b) cRNA molecules and incubated at 31, 37 or 41 °C for the indicated periods of time in the presence of heparin before analysis by non-denaturing PAGE and autoradiography. (c) The amounts of polymerase-template complexes were quantified by densitometry and expressed as the fraction of complex remaining compared to T_0_. The average and range of two independent experiments is plotted.

Given our observations of heat stable transcriptionally active polymerase and near normal accumulation of plus-sense RNA species coupled with a specific defect in vRNA synthesis, we hypothesised that the interaction of polymerase with its cRNA template might be particularly temperature sensitive [52,53]. The interaction of trimeric polymerase with promoter RNAs was therefore tested at the appropriate temperatures using a bandshift assay based on recombinant polymerase expressed from vaccinia virus and short synthetic v- and cRNA panhandle RNAs [24]. When nuclear extracts containing the influenza virus polymerase were tested, a low electrophoretic mobility radiolabelled complex was observed for both v- and c-RNA (Fig. 7, lane 3). No complex was observed when nuclear extracts containing bacteriophage T7 RNA polymerase were examined (Fig. 7A, B; lanes 1 and 2). To measure the dissociation rate of the polymerase-panhandle interaction, complexes were allowed to form at room temperature, then heparin was added and the binding reactions incubated at 31, 37 or 41°C for varying lengths of time before analysis. Heparin effectively prevents the initial binding of the polymerase to the template RNA, thus it can be used to prevent reassociation of the polymerase during a dissociation experiment [24,54]. At 31°C, polymerase-panhandle complexes were stable over the time-course examined, for both v- and cRNA (Fig. 7A, B; lanes 12–15). At 41°C, polymerase-promoter RNA complexes were unstable, with both v- and cRNA complexes showing significant amounts of dissociation by 40 minutes (Fig. 7A, B; lanes 4 – 7). However, polymerase-cRNA complexes were clearly less stable than the equivalent vRNA structure. Replicate experiments determined the t1/2 for dissociation of the polymerase at 41°C to be around 10 minutes for cRNA but greater than 40 minutes for vRNA (Fig. 7C). Furthermore, when dissociation was examined at 37°C, polymerase-vRNA complexes were essentially stable but nearly half of the polymerase molecules initially bound to cRNA had dissociated after 40 minutes (Fig. 7A, B; lanes 8–11, Fig. 7C). These data indicate that the interaction of the influenza virus polymerase with cRNA promoter is indeed significantly less stable at elevated temperatures than the interaction with the vRNA counterpart. This suggests that negative strand synthesis fails at high temperature simply because the polymerase is unable to productively interact with the appropriate promoter.

**Figure 8:**
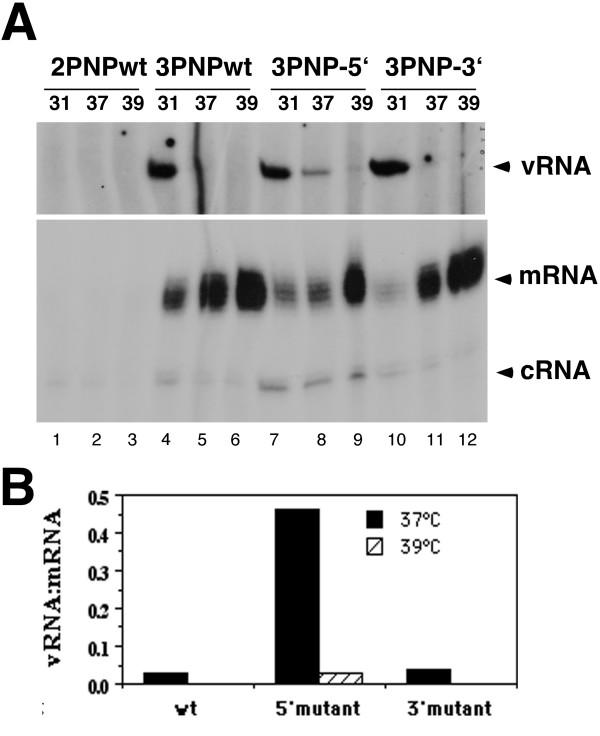
**Effect of cRNA promoter mutations on temperature sensitive vRNA synthesis**. 293T cells were transfected and incubated as described for figure 2, using either a wild type pPol-I(+)NSCAT or two promoter mutants that transplant a vRNA-like base pair into the cRNA promoter; mutants 3'U-8'A (5'-end) or 3G-8C (3'-end). **A**. Total RNA was harvested and analyzed by primer extension as described before to detect virus-derived CAT RNA species. **B**. Radiolabelled products for m- and vRNA were quantified by densitometry. Mean ratio of vRNA:mRNA at 37°C and 39°C from two independent experiments is plotted.

To further analyse this hypothesis, we tested whether mutations in the cRNA promoter that render it more vRNA-like would rescue normal viral genome replication at high temperature. For this, we utilised two mutants in which either the 5'-end (3'U-8'A) or the 3'-end (3G-8C) of the cRNA promoter was altered [55]. 293T cells were co-transfected with plasmids that expressed the three polymerase proteins, NP and wild-type (WT) or mutant model cRNA segments containing a CAT gene. Cells were then incubated either at 31°C, 37°C or 39°C for three days and viral RNA accumulation analyzed. No products were observed from cells that were transfected with all plasmids except PB1 (Fig. 8A, lanes 1–3). When cells expressed the complete set of RNP polypeptides and the wild type positive-sense model segment, the amount of replicative RNA products (c- and vRNA) decreased sharply at 37°C, whereas accumulation of mRNA increased as the incubation temperature was raised from 31°C to 39°C (Fig. 8A, lanes 4–6), as shown before. The same pattern of temperature dependent viral RNA synthesis was observed when a cRNA segment with the 3G-8C mutation in its 3'-end was transfected (lanes 10–12). When the 5'-end 3'U-8'A mutant was used, lower levels of mRNA and higher of cRNA were observed compared to the wild type segment (Fig. 8A, compare lanes 7–9 to 4–6), as previously described [55]. Significantly however, synthesis of c- and vRNA was less affected by increasing temperature when using the 5'-end mutant and mRNA synthesis did not increase with temperature as dramatically as when wild type cRNA was used (lanes 7–9, compare to lanes 4–6). Radiolabeled products obtained with the three different promoters (WT, 3'U-8'A and 3G-8C) were quantified by densitometry in replicate experiments. When cells were incubated at either 37°C or 39°C, the ratio of vRNA to mRNA increased by around 10 fold when cRNA segment was mutated in its 5'-end, compared to both WT and 3'-end mutated promoter (Fig. 8B). Thus the balance between transcription and replication catalysed by WT polymerase becomes less temperature sensitive when supplied with a hybrid cRNA promoter containing mutations in the 5'- but not 3'-end. Since the primary interaction of the polymerase with the cRNA promoter occurs with the 5'-end of the structure [53], this finding supports our hypothesis that temperature sensitive genome replication results from heat-labile polymerase binding to the plus-strand template.

## Discussion

Here, we show that temperature dramatically affects the balance between transcription and replication of the influenza virus genome, with high temperature in particular favouring mRNA production over vRNA synthesis (Figs. 1 and 2). This effect was observed for both recombinant and authentic viral RNPs, for three strains of viral RNPs and in two different cell types, suggesting it is a general phenomenon for mammalian-adapted influenza A virus replication. The down regulation of vRNA synthesis at high temperature did not result from a deficiency of a viral polypeptide necessary for genome replication as no significant reduction was seen in the quantities of any of the viral proteins (Figs. 1, 2, 4 and data not shown). Although a recent report has suggested that NS1 is a necessary co-factor for vRNA synthesis [56], we found no defect in NS1 translation (Fig. 1C) and NS1 is clearly not required for replication in the recombinant system (Fig. 2). It was necessary to address the hypothesis that cellular heat shock proteins were responsible for the reduction in vRNA synthesis, as previous reports have indicated that hsp90 interacts with both PB2 and PB1-PA complexes at 37°C [26,30] and that hsp70 binds NP at 37°C and 41°C [41]. In vitro at 30°C, hsp90 stimulates ApG-primed transcription of an exogenous template added to virion-derived RNPs, while hsp70 has little effect [30]. An interaction of hsp70 with NP has however been proposed to block M1-RNP interactions and consequently inhibit RNP nuclear export [41]. However, the temperature dependent inhibition of genome replication showed no correlation with the amount of hsp70 and hsp90 present in cells. Heat shock prior to infection to induce hsp synthesis did not alter viral RNA synthesis at 37°C (Fig. 3). Furthermore, inhibition of hsp70 induction with quercetin did not rescue vRNA synthesis at 41°C (Fig. 4A). Similarly, geldanamycin-mediated inhibition of the chaperone function of hsp90 failed to rescue vRNA synthesis at 41°C (Fig. 4B). Hirayama et al. (2004) showed that in infected MDCK cells prostaglandin A1 (PGA) blocks NP nuclear export at 37°C and induces hsp70 expression without inhibiting viral gene expression. However, in 293-T cells we found that PGA inhibited all forms of viral RNA synthesis and viral protein expression (data not shown). Other studies have also noted inhibition of influenza virus gene expression by PGA and other prostaglandins and related compounds that induce hsp70 as well as a variety of other stress-related polypeptides [57,58]. Overall therefore, although it is difficult to rule out categorically, we find no evidence in favour of the hypothesis that cellular hsp70 and/or 90 mediate the inhibition of viral genome replication at high temperature.

Two observations relating to the effects of hsps on virus replication are worthy of further comment. Firstly, while previous observations [41,49] regarding the inhibition of NP nuclear export at 41°C were confirmed (Fig. 3), RNP formation was found to be abnormal, whereas the study of Sakaguchi et al (2003) [49] noted unaltered NP sedimentation behaviour on glycerol density gradients. The discrepancy between the two studies may result from the different gradient fractionation methods employed and the fact that Sakaguchi et al. looked solely at NP sedimentation and not RNA and polymerase protein sedimentation in addition to NP (reported here), which provides a more rigorous test of RNP formation (Fig. 5). Therefore, another contributing factor to the failure of NP nuclear export at 41°C is that RNP formation is much reduced. Secondly, the observation that moderate concentrations of geldanamycin inhibit PB2 accumulation at 37°C and 41°C, which is consistent with prior data indicating an interaction between this viral protein and hsp90 [26,30] raises the possibility that geldanamycin may have antiviral activity against influenza A as well as a variety of other viruses [59-62].

**Figure 9:**
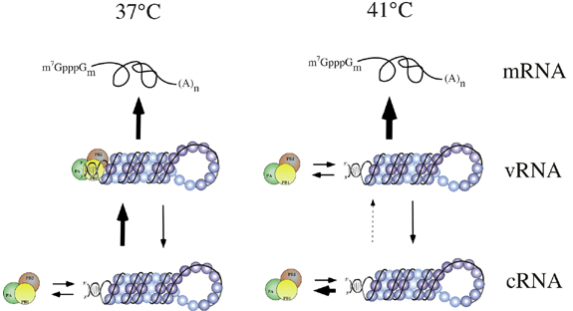
**Cartoon model of the effect of temperature on polymerase-RNP interactions and viral RNA synthesis**. The size of arrow represents the relative rate of each process. At 37°C, polymerase-vRNA complexes are stable and catalyse the synthesis of mRNA and low amounts of cRNA. Polymerase-cRNA complexes show a moderate rate of dissociation and reassociation that permits high levels of vRNA synthesis. At 41°C, polymerase-vRNA complexes are less stable but still permit high level mRNA synthesis and near normal cRNA synthesis. However, the high rate with which polymerase-cRNA complexes dissociate does not permit efficient vRNA synthesis.

Although temperature sensitive interactions with cellular components other than hsp proteins cannot be ruled out it is appropriate to consider explanations for the loss of vRNA synthesis at high temperature that are intrinsic to the viral components themselves. Temperature-shift cycloheximide block experiments showed that the key determinant that lead to a failure of vRNA synthesis was high temperature during RNA synthesis, and not during polymerase translation, and that polymerase synthesised at 41°C retained the ability to synthesise mRNA at 37°C and 41°C (Fig. 6). Furthermore, in vitro, there is a clear difference in the thermal sensitivity of polymerase-vRNA and polymerase-cRNA complexes (Fig. 7). This finding suggests a simple model that explains our results. At 41°C the polymerase shows a slow rate of dissociation from vRNA (Fig. 7). At least a proportion of the polymerase that dissociates from v- or cRNA at 41°C has been found to be capable of rebinding to its template (data not shown). We hypothesise therefore that an equilibrium exists between polymerase and vRNA at 41°C that permits substantial amounts of mRNA and cRNA synthesis (Fig 9). However, the far higher rate of dissociation from cRNA at elevated temperature biases the equilibrium further away from mature cRNP formation and leads to a proportionally greater defect in vRNA synthesis (Fig. 9). In support of this hypothesis, a cRNA promoter mutation that makes it more similar to the vRNA structure reduced the effect of high temperature on vRNA synthesis (Fig. 8).

The two-fold reduction in cRNA accumulation at 41°C might result from a reduced rate of synthesis, or from increased degradation of "naked" cRNPs, consistent with a recently suggested hypothesis for how cRNA synthesis is regulated [21]. Increased degradation rates of RNPs may explain the apparently greater thermal sensitivity of recombinant RNPs (Figs. 2 and 8) because of the much longer timeframe of these assays (3 days compared to ≤ 8 h). The apparent paradox between increased levels of mRNA synthesis in the face of reduced vRNA levels may result from a higher rate of transcription of a reduced number of template molecules. This might reflect a Q_10 _effect of the higher temperature but may also result from loss of normal regulatory patterns (such as nuclear export of vRNA) during viral transcription. Consistent with the latter possibility, previous studies have noted substantial levels of mRNA transcription from a limited number of input vRNA templates when genome replication is blocked by cycloheximide [11,12,63].

At 37°C our finding that there is a negligible dissociation rate between polymerase and vRNA is consistent with the currently accepted mechanism for polyadenylation. In this hypothesis, the polymerase remains bound to the 5'-end of vRNA throughout mRNA transcription, thus forcing non-processive copying of a poly(U) stretch adjacent to the polymerase-binding site [6,7,54,64,65]. In light of recent research indicating that RNPs localise to specific regions within the nucleus [66,67], a mechanism whereby vRNA molecules can be reiteratively copied without release of the polymerase seems likely to increase the efficiency of template usage. The significantly higher dissociation rate for polymerase-cRNA complexes at 37°C reflects a weaker interaction of the polymerase with the 5'-end of cRNA. This is consistent with the need to synthesise only full length copies from this template which presumably requires a template release step for each cycle of vRNA synthesis (Fig. 9). This is perhaps surprising given that large amounts of vRNA molecules are manufactured from a limited number of cRNAs whose synthesis apparently ceases early in infection [5,68]. However, this quantitative difference in how the polymerase interacts with c- and vRNA promoters is consistent with recent research suggesting a qualitative difference in the mechanism used to initiate unprimed (replicative) transcription from the two strands [69].

## Conclusion

Human influenza A virus, genome RNA synthesis is inhibited at high temperature, with transcription strongly favoured over replication. This temperature effect is due to the differential stability of negative and positive sense polymerase-promoter complexes, which is accentuated at high temperature (fig. 9). These findings have implications for the mechanisms that control normal viral RNA synthesis. Furthermore, as temperature is a notable physiological difference between avian and mammalian host systems, such considerations may have significance for the influence of avian virus-derived polymerase genes on host range and pathogenesis.

## Methods

### Cells, virus, plasmids, antibodies and other compounds

Human embryonic kidney 293T cells and Madin-Darby canine kidney (MDCK) cells were cultured in Dulbecco's modified Eagle's medium supplemented with L-glutamine, penicillin, streptomycin and 10% fetal calf serum (FCS). Influenza virus strain PR8 was propagated in 10-day-old embryonated eggs as described previously [70]. Infections were carried out at a m.o.i. of 5 and generally harvested at 5 h.p.i.

Plasmids pcDNA-PB2, -PB1, -PA and -NP are described elsewhere [20,44]. pPolI(-)NS.CAT.RT, containing an antisense CAT gene flanked by the non-coding sequences of influenza A/WSN/33 virus segment 8, under control of a human RNA polymerase I promoter (Pol-I) and upstream of a hepatitis δ ribozyme sequence, was generously provided by Ervin Fodor (University of Oxford). A similar positive-sense CAT reporter [pPol-I(+)NS.CAT] has also been previously described [20]. Plasmids pPOLI-cCAT-RT wild type, 3'U-8'A mutant (C-G to U-A in 5'-end) and 3G-8C mutant (A-U to G-C in 3'-end) were kindly provided by G. Brownlee [55]. Plasmids p5'3'v and p5'3'c contain the T7 RNA polymerase promoter immediately upstream of model vRNA and cRNA template sequences such that linearization with MboII and transcription with T7 RNA polymerase leads to the synthesis of run-off transcripts with the sequence: **AGUAGAAACAAGGGUGUUUUUU**CCCGGGAAUUCGGAUCCA**CACCCUGCUUUUGCU**and **AGCAAAAGCAGGGUG**UGUGGAUCCGAAUUCCCGGGU**AAAAAACACCCUUGUUUCUACU**, where the underlined bold sequences correspond to the terminal regions of segment 8 (NS1) vRNA and cRNA respectively.

Nuclear extracts of HeLa cells co-infected with vaccinia recombinants expressing the PB1, PB2 and PA subunits of the PR8 polymerase (Vac3P) were prepared as described previously [54].

Rabbit polyclonal antisera to influenza A virus RNP and anti-PR8 PB2 (MBP-PB2-C), PB1 (PB1-F1) and PA (PA-F3) have been described previously [19,71]. Mouse monoclonal anti-PB1 (aa 44–69) was kindly provided by Dr. Mark Krystal (Bristol-Myers Squibb). Antibodies to β-actin, Lamin associated polypeptide 2 (LAP-2) and clathrin heavy chain were purchased from Sigma-Aldrich, Biosciences Pharmingen and Santa Cruz, respectively. Antibody to hsc72/hsp70 was supplied by BD Bioscience. Anti-rabbit IgG antibody conjugated to fluorescein isothiocyanate was supplied by DAKO A/S and Alexa 594-conjugated anti-mouse IgG antibodies by Molecular Probes. HRP-conjugated anti-rabbit or mouse IgG was purchased from Amersham Biosciences.

Quercetin was purchased from Calbiochem and cycloheximide and geldanamycin from Sigma. Drugs were dissolved in dimethylsulfoxide (DMSO) and stored at -20°C, except quercetin, which was stored at 4°C.

### Influenza virus gene-expression assay

To reconstitute RNPs, 1 × 10^6 ^293-T cells per 35 mm well in 1 ml complete medium were transfected in suspension with 0.25 μg each of pcDNA-PB1, -PB2, -PA and -NP and 50 ng of either pPolI(+) NS.CAT, pPolI(-)NS.CAT.RT, 3'-8' mutant or 3–8 mutant, using cationic liposomes (Lipofectin; Gibco-BRL). The total amount of DNA in each transfection mix was normalized by the addition of pcDNA3 vector. Transfected cells were incubated for three days before harvest [20].

### Primer-extension analysis of viral RNA

Total cellular RNA was isolated using a commercial kit (SV Total RNA Isolation System; Promega) according to the manufacturer's instructions. After spectrophotometric quantification and normalization, RNA was reverse-transcribed using avian myeloblastosis virus reverse transcriptase (Promega) and the appropriate DNA oligonucleotides that had been 5' end-labelled with [γ-^32^P]ATP as previously described [20]. Radiolabelled products were detected by autoradiography and quantified by densitometry using NIH Image software [72]. The oligonucleotides used were 5'-GAACTGAGCAACCTTGCG-3' (for detection of vRNA-sense segment 1), 5'-GTACTTCTTGATTATGGC-3' (positive-sense segment 1), 5'-TCCAGTATGGTGGAGGC-3' (vRNA-sense segment 2), 5'-GTATCCTGTTCCTGTCCC-3' (positive-sense segment 2), 5'-CAAATAACATTTATGCAAGCC-3' (vRNA-sense segment 8) and 5'-TTAGGGATTTCTGATCTCGGC-3' (positive-sense segment 8). The oligonucleotides used to detect segments, 5, 7 and CAT have been previously described [20].

### Protein analysis

For Western blots, cell lysates were separated by SDS-PAGE and transferred to nitrocellulose. Blots were probed with primary followed by secondary antibodies conjugated to horse-radish peroxidase (DAKO) and developed by chemiluminescence (ECL reagent; Amersham Biosciences). Immunofluorescence assays were carried out as previously described [70,44]. For metabolic labelling of proteins, 293T cells were washed twice with methionine-free medium for 5 min. and then incubated for 2 h with 60 nCi/μl of ^35^S-methionine For immunoprecipitation, 4 × 10^5 ^cells were labelled at 4 hpi as described above, then harvested in 200 μl IP buffer (50 mM Tris-HCl, pH7.6, 100 mM KCl, 5 mM MgCl_2_) containing 0.1% NP40 before being subjected to immunoprecipitation as previously described [73].

### Velocity gradient centrifugation

8 × 10^6 ^293T cells were lysed with 1 ml of buffer containing 0.5% Triton X-100, 10 mM NaCl and 20 mM Tris-HCl (pH7.6), treated with 10 μg/ml Dnase I (Sigma), 5 mM MgCl_2_, 10 mM vanadyl ribonucleoside complex (New England Biolabs) for 15 min at 37°C and clarified at 3,000 rpm for 5 min at 4°C in a minicentrifuge. The supernatant was layered on top of a 12 ml continuous 5–20% (w/v) sucrose gradient in 20 mM Tris-Cl pH7.6, 50 mM NaCl, 0.1 mM EDTA and centrifuged at 100,000 g_av _for 13 h [73]. Fractions were then collected and analysed for their protein and RNA content by western blot and primer extension respectively. For this, protein was concentrated by precipitation with methanol-chloroform [74] and RNA was isolated by phenol:chloroform extraction and ethanol precipitation.

### Polymerase binding assays

The preparation of RNA transcripts labelled internally with [α-^32^P] GTP and the binding assay protocol have been described previously [54]. 150 μl binding reactions containing 1.5 × 10^5 ^cpm of labelled probe (5'3'v or 5'3'c) were incubated with 30 μg of Vac 3P extract for 10 min at 22°C to allow the polymerase to bind to the RNA. Heparin was then added to a final concentration of 5 mg/ml, sufficient to inhibit all subsequent binding of the polymerase and displace non-specific RNA binding of other proteins in the extract. The binding reactions were then aliquoted into 12 separate tubes and incubated at either 31°C, 37°C or 41°C. At regular intervals, one tube from each temperature was removed and placed on ice until the completion of the time course. The samples were then analysed by non-denaturing electrophoresis and autoradiography as described previously [54].

## Competing interests

The author(s) declare that they have no competing interests.

## Authors' contributions

RMD performed the majority of the experiments, analysed the results and drafted the manuscript. AEM performed some of the transfection experiments. MJA designed and validated aspects of the primer extension assay. EM generated some of the antibodies used and provided technical assistance. LT performed the band-shift experiments and helped in analysing the results. PD oversaw the project design and completion, provided the resources, aided in analysing the results and helped write the manuscript. All authors read and approved the final manuscript.
